# Effects of Slaughter Age on Myosin Heavy Chain Isoforms, Muscle Fibers, Fatty Acids, and Meat Quality in *Longissimus Thoracis* Muscle of Tibetan Sheep

**DOI:** 10.3389/fvets.2021.689589

**Published:** 2021-10-26

**Authors:** Gaoliang Bao, Xiu Liu, Jiqing Wang, Jiang Hu, Bingang Shi, Shaobin Li, Yuzhu Luo

**Affiliations:** Gansu Key Laboratory of Herbivorous Animal Biotechnology, Faculty of Animal Science and Technology, Gansu Agricultural University, Lanzhou, China

**Keywords:** muscle fiber, meat quality, fatty acid, MyHC isoform, Tibetan sheep

## Abstract

Tibetan sheep is one of the dominant livestock at Qinghai-Tibet Plateau, which is the main food source of local people. In order to investigate the effect of slaughter age on meat quality, fatty acid profile and expression of myosin heavy chain (MyHC) isoform genes were analyzed in Tibetan sheep. A total of 24 Tibetan sheep including 4 months old (4 m), 1.5 years old (1.5 y), 3.5 years old (3.5 y), and 6 years old (6 y) were randomly selected. The results indicated that the MyHC IIx and MyHC IIb mRNAs increased with age, whereas MyHC IIa mRNA decreased. MyHC I mRNA was highest at 3.5 y. There were differences in the muscle fiber types of Tibetan sheep at different ages. Intramuscular fat (IMF) was highest at 1.5 y, the pH_45min_ and pH_24h_ value of 6 y sheep were lower than the other groups, the shear force increased with age (*p* < 0.05), and drip loss increased with age (*p* < 0.01). Tibetan sheep at 1.5 y had lower saturated fatty acid (SFA) contents and higher monounsaturated fatty acid (MUFA) contents (*p* < 0.05). Different muscle fiber types influence the meat quality and fatty acid composition of Tibetan sheep with increasing age. These results demonstrated the effect of age on meat quality of Tibetan sheep through regulation of expression of the MyHC isoforms which changed the myofiber types, and 1.5 y Tibetan sheep meat was more suitable for a healthy human diet.

## Introduction

Consumers, especially from developed countries, are more health conscious and pay more attention to nutritional and physicochemical qualities of the meat products they consume (1). Nutritional and physicochemical qualities are affected by various factors including the slaughter age (2), breed, sex, and diet of the animal (3). Animal age is an important influencing factor that can affect both the meat quality and fatty acid profile (4). The intramuscular fat (IMF) content accumulates with increasing age, improving water holding capacity and juiciness (5). Abhijith et al. have found that meat tenderness of boer goats decreased with increasing slaughter age (6). Guo et al. demonstrated that the shear force of pork increased with slaughter age, while IMF content first decreased and then increased (7). The Tibetan sheep is the most numerous livestock (>50 million) on the Qinghai-Tibet Plateau and is also the main meat source for local people (8). Tibetan sheep is well-known for its highly nutritious, superior meat quality and strong resistance to harsh environments. However, effects of slaughter age on fatty acids and meat quality of Tibetan sheep remains unknown.

The energy level of the muscles of animals at slaughter affects the energy metabolism of the muscle during the post-mortem period, causing differences in meat quality (9). Glycolysis is a very important energy pathway in cells during the post-mortem period, and the glycolysis rate is influenced by muscle fiber type (10). There are mainly four muscle fiber types in adult mammalian skeletal muscle: MyHC I (types I), MyHC IIa (types IIa), MyHC IIb (types IIb), and MyHC IIx (types IIx) (11, 12). Moreover, the structure, function, and metabolism of the four muscle fiber types are different. The myosin heavy chain (MyHC) gene directly regulates the type of muscle fibers through gene expression and has an important impact on meat quality (10). Besides, the proportions of different muscle fibers change with age in humans, and this transformation also occurs in the muscles of animals, such as pigs, chickens, sheep, and mice (13). Different types of muscle fibers contain different contents of myoglobin, mitochondria, glycogen, and fat, affecting their metabolic characteristics, which will lead to differences in muscle quality including in terms of muscle tenderness, color, and IMF (14). Previous studies have underlined the relationship between muscle fiber types and meat quality and fatty acid. Renand et al. found that IMF content is an important factor in determining eating quality including tenderness, juiciness, and flavor, and IMF content is positively correlated with meat tenderness (15). Alasnier et al. demonstrated that the percentage of type I muscle fibers in beef was positively correlated with IMF content (16). Tenderness is an important indicator for evaluating meat quality, which is the key factor affecting consumer purchasing and market acceptance (17). Therkildsen et al. and Ryu et al. demonstrated that the proportion of type I muscle fibers was positively correlated with the juiciness and flavor of meat but negatively correlated with drip loss and brightness of the flesh color (18, 19). Conversely, the more percentage of type IIB muscle fibers, the drier and harder the meat (20). Joo et al. found that muscle fiber type composition is an important factor influencing fatty acid composition in Hanwoo muscle (21). Han et al. found that appropriate fiber level addition in diets could improve meat quality through regulation the expression of myofiber types (20). However, effects of muscle fiber types on fatty acids and meat quality of Tibetan sheep remains unclear.

Therefore, the objective of the present study was to determine the changes of muscle fiber types with increasing age to evaluate the more suitable slaughter age of Tibetan sheep by determining the meat qualities and fatty acid. The associations of muscle fiber types and expression of MyHC isoform genes on meat quality of the *Longissimus thoracis* (LT) in Tibetan sheep at four growth stages (4 m, lamb; 1.5 y, young sheep; 3.5 y, adult sheep and 6 y sheep) were analyzed.

## Materials and Methods

### Ethics Statement

This animal study was reviewed and approved by the Faculty Animal Policy and Welfare Committee of Gansu Agricultural University (Ethic approval file No. GSAU-Eth-AST-2021-001).

### Animals and Muscle Sampling

A total of 24 Tibetan sheep ewes representing four stages of growth (4 m, 1.5 y, 3.5 y, and 6 y) with each stage having six individuals were randomly selected from the same flock of Haiyan County, Qinghai Province, China (3,500 m above sea level). All sheep had the same nutrition and were raised under the same environmental conditions with natural light and free access to food and water. All these sheep were exsanguinated humanely according to Islamic customs, peeled, and split down the midline according to standard operating procedures. Each muscle sample of *Longissimus thoracis* at the 12th and 13th rib was collected from the carcass immediately after slaughter; after rinsing with saline and de-RNAase water, RT-qPCR samples were collected in cryotubes and stored in liquid nitrogen immediately. Meanwhile, two pieces of the left LT muscles between the 12th and 13th ribs were sampled, one of them snap-frozen in liquid nitrogen for ATP staining, and another one was fixed in 4% paraformaldehyde (PFA) for HE staining. Carcasses were then chilled at 4°C for 24 h for further meat quality analysis.

### Meat Quality Measurements

The pH of the LT muscle between the 12th and 13th ribs was measured at 45 min (pH_45min_) and 24 h (pH_24h_) after slaughter using a portable pH meter (Testo 205 portable waterproof pH, Testo Instruments International Trading Ltd., Shanghai, China) by inserting directly into the samples. The electrode was calibrated with pH 4.00 and pH 7.00 buffer before determination. Each sample was measured three times, and the average value was used for statistical analyses. Meat color was measured using a Minolta chromameter (CR-300; Minolta Camera Co., Osaka, Japan) calibrated against a standard white tile (8 mm diameter aperture, D65 illuminant and 10° standard observer angle) at 45 min and 24 h after slaughter at three positions on the surface of the cross-section of LT after exposing the sample to air for 30 min at 4°C. Results were expressed as lightness (L^*^), redness (a^*^), and yellowness (b^*^) (22). For cooking loss analysis, 300-g samples were steamed in a water bath to a core temperature of 70°C. The cooking loss was calculated as the percentage of weight change before and after cooking (23). The shear force was measured at the 12th to 13th ribs according to the method of Honikel (24). Forty-eight hours after slaughter, the samples were cooked within a plastic bag set in a water bath at 75°C until the internal temperature of the sample reached 70°C; it was then cooled down to room temperature. Five slices of meat each with a diameter of 1.27 cm were cut from each sample with a cylindrical core drill, the slices being cut perpendicular to the fiber direction with a shearing device (C-LM3B; Runhu Instrument Co., Ltd., Guangzhou, China). Following the method of Latorre et al. (25), approximately 25 g of muscle from the 12th and 13th ribs were used to analyze drip loss. The muscle samples were trimmed to 2 × 3 × 5 cm along the length of the fiber and suspended in an inflatable plastic bag, then stored at 4°C for 24 h. The drip loss was calculated as the proportion of lost weight compared to the initial weight. The content of IMF in the LT at the 12th to 13th ribs was measured by the Soxhlet extraction method using a solvent (petroleum ether) and expressed as weight as a percentage of wet muscle tissue (26), with three replicates for each sample.

### Fatty Acid Profile

Fatty acids in the LT of Tibetan sheep were determined according to the method by Juárez et al. (27) with several modifications. The meat samples were ground in liquid nitrogen; 1.0 g of the ground sample was Heated by a water bath with 0.7 ml of 10 mol·L^−1^ KOH solution and 5.3 ml anhydrous methanol (chromatographically pure), at 55°C for 1.5 h. The test tube and contents were then cooled to room temperature, mixed with 0.58 ml 12 mol·L^−1^ H_2_SO_4_ solution, and the free fatty acid was methylated and placed in a water bath at 55°C for 1.5 h. It was then cooled to room temperature, 3 ml n-hexane was added, then transferred to a centrifuge tube and centrifuged at 3,000 rpm for 5 min. The supernatant was filtered into short thread vials (Beijing Labgic Technology Co., Ltd., Beijing, China) using a 0.22 μm nylon syringe, and stored at −20°C for fatty acid methyl esters (FAMEs) analysis. FAMEs were separated and quantified on a Shimadzu GC2010 Plus with a Flame Ionization Detector (FID), a split injector, and an AOC-20i auto-injector. A Restek FAMEWAX capillary column (0.25 mm × 30 m × 0.25 μm) was used. The initial oven temperature was 140°C for 5 min, then increased to 200°C at a rate of 2°C per minute, and then to 230°C at a rate of 6°C per minute, then temperature was maintained for 20 min. FAME peaks were identified and quantified by the comparison of their retention times with those produced from a mixture of 37 FAME standards (Supelco 47,885-U; Sigma-Aldrich, St. Louis, MO, USA), and then serially diluted to five concentrations ranging from 10 to 0.625 g L^−1^. Fatty acid analysis was conducted in triplicate for each sample.

### HE Staining

HE staining analysis was conducted as described by Zhang et al. (28). 1 × 2 × 0.5 cm sample was soaked to 4% paraformaldehyde solution for 24 h. After formalin fixation and dehydration, samples were encased in paraffin wax and sliced into 4-μm-thick histological sections. Tissue sections were stained with hematoxylin–eosin, and images were collected using an upright microscope (IX71; Olympus Microsystems Ltd., Tokyo, Japan). Then, at least 100 muscle fibers were randomly selected for measurement of their diameter using Image-Pro Plus 6.0 (Media Cybernetics Inc., Rockville, MD, USA) image analysis software.

### Myosine ATPase Staining

The contractile fibers type (Type I, IIA, IIx, and IIB) of LT muscles were identified using the myosin ATPase staining technique as previously described by Sen et al. (29) with some modifications. Acid pre-incubation ATP enzyme histochemical staining was undertaken. After the sections were dried, acid buffer (0.2 mol/L sodium acetate 49 ml, 0.2 mol/L glacial acetic acid 45 ml, pH 4.63) was added for 10 min. The sections were then removed and incubated with acid ATPase solution. This involved adding 5 ml of ATPase working solution, then 0.6 g adenosine disodium triphosphate and 15 ml of distilled water to dilute, resulting in a pH value of 9.4–9.5. These sections were then washed two to three times for 2 min each time. Acid ATPase incubation solution was added and the sections were incubated at 37°C for 2 h. The working solution was then removed, the sections were then placed in 1% calcium chloride solution for 6 min, the solution was then removed, and the sections were placed in 2% cobalt chloride solution and then washed three times with distilled water, for 5 min each time. Ammonium sulfide solution was added to the sections to develop their color, for about 1 min. They were then washed with tap water three times and transparently mounted using ethanol dehydrated xylene.

The succinate dehydrogenase staining technique described by Nachlas et al. (30) was used to identify the metabolic types (oxidized and glycolytic) of the LT muscle fibers. The muscle fibers were observed using a microscope (×100, Olympus BX61; Olympus Corporation, Tokyo, Japan) and connected to an image capture system (Clemex image analysis software; Vision Lite, Montreal, Canada). Image-Pro Plus 6.0 (Media Cybernetics Inc.) analysis software was used for data analysis of type I, type IIA, and type IIB fibers. Type I fibers are usually dark brown, type IIA + IIX fibers have the lightest coloration, and the color of type IIB fibers are between type I and IIA + IIX types.

### Quantitative Real-Time PCR Analysis

Total RNA was extracted from LT muscle samples of Tibetan sheep using Trizol Reagent (Shanghai Yuanye Biotechnology Co., Ltd., Shanghai, China). The experimental operation was carried out according to the product instructions. The purity and concentration of the extracted RNA were measured by an ultraviolet spectrophotometer, and then it was stored at −80°C. The Prime Script™ RT reagent Kit with gDNA Eraser was used for cDNA reverse transcription. The reverse-transcribed cDNA was stored at −20°C for the next analysis.

The NCBI database and Primer Premier 5 software (Premier Biosoft, Palo Alto, CA, USA) were used to design primers to amplify the MyHC isoform genes and a housekeeping gene for qPCR. The primer sequences and PCR conditions are listed in Table 1. cDNA was used as a template and the SYBR Green Pro Taq HS qPCR Kit (Accurate Biology, Hunan, China) was used for RT-qPCR. The thermal cycling parameters were as follows: an initial denaturation step at 95°C for 10 min followed by 40 cycles of denaturation at 95°C for 15 s and annealing and extension at 60°C for 1 min. The expression of different MyHC mRNAs were determined as 2^−ΔΔCt^, where ΔCt was the difference in Ct between the MyHC and glyceraldehyde 3-phosphate dehydrogenase (GAPDH). The relative amounts of MyHCI, IIa, IIx, and IIb were expressed as the percentage of total MyHC transcripts.

**Table 1:** Primer sequences and annealing temperatures used for RT-qPCR.

**GenBank accession No**.	**Gene**	**Primer sequence (5^**′**^-3^**′**^)**	**Annealing temperature (^**°**^C)**
XM_004010325.3	MyHC I	F: GCAAGAAGAGGAGTGAGGCA	60
		R: GGCAGCAATGACCGCAAA	
XM_027974884.1	MyHC IIa	F: CTGAGGAGGCTGAGGAACA	60
		R: TCAGGACACGATCACTCTTCA	
XM_027974882.1	MyHC IIx	F: AACAACTTCCAGAAACCCAAAC	60
		R: GTACAGCCCGACCACCGT	
XM_027974883.1	MyHC IIb	F: TGAGGCAACAAAGAATCTTA	60
		GAAAC	
		R: AAGTGGAGCTGAGTGTCCTTC	
NM_001190390	GAPDH	F: GTCGGAGTGAACGGATTTGG	60
		R: ACGATGTCCACTTTGCCAGT	

### Statistical Analysis

All statistical analyses of the difference were performed using one-way analysis of variance (ANOVA) by IBM SPSS 22.0 (SPSS Inc., Chicago, IL, USA). Significance among the groups was investigated by Duncan's multiple range tests. Data are shown as the means ± standard error (S.E). Pearson correlation analysis was used to analyze the relationship between meat quality traits and muscle fiber characteristics. GraphPad Prism v6.04 Software (GraphPad Software Inc., San Diego, CA, USA) was used to analyze the dynamics and plot the graphs. Each experiment was replicated at least thrice.

## Results

### Muscle Development at Different Growth Stages of Tibetan Sheep

**Figure 1:**
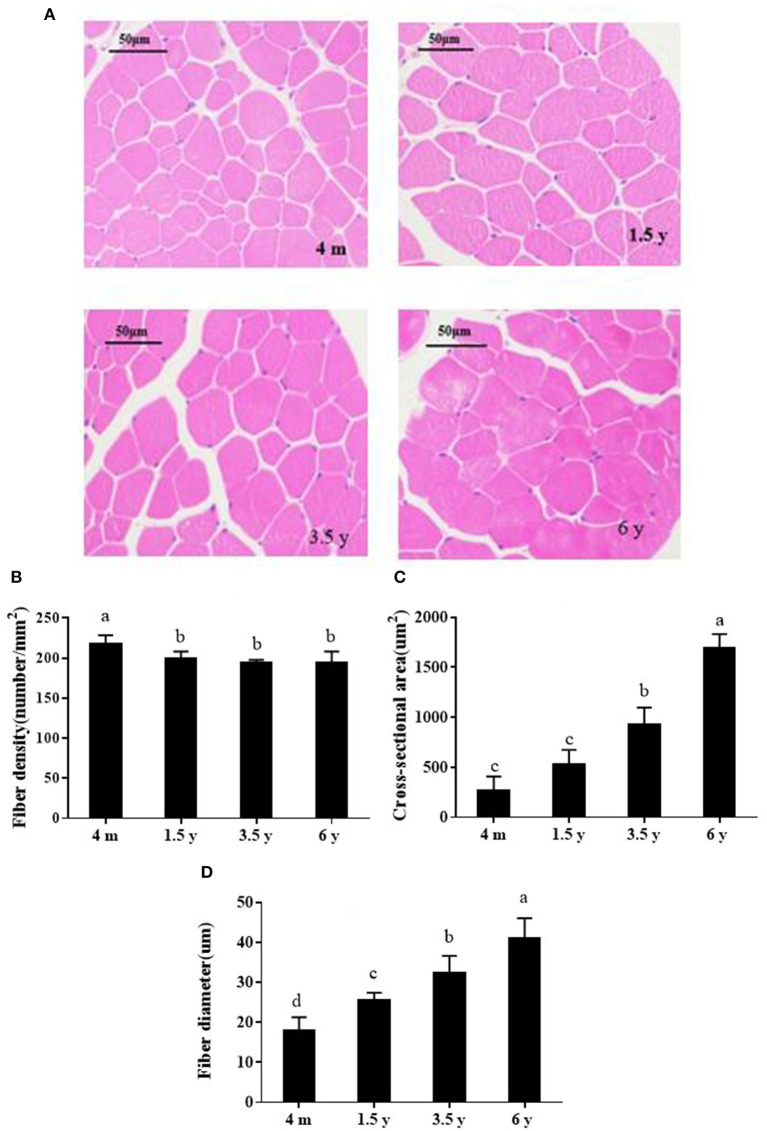
**(A)** HE-stained photos of the muscle fibers of Tibetan sheep at different growth stages (×200). **(B–D)** Show muscle fiber density, area and diameter, respectively. Data are shown as the means ± S.E. Different letters (a–d) indicate significant differences between different growth stages (*p* < 0.05). 4 m, 4 months old; 1.5 y, 1.5 years old; 3.5 y, 3.5 years old; 6 y, 6 years old.

HE staining was undertaken to analyze the muscle development of Tibetan sheep at different growth stages (Figure 1), including muscle fiber diameter, area, and density. The muscle fiber density of 4 m was significantly larger than other groups (*p* < 0.05), and there was no significant difference among 1.5, 3.5, and 6 y (*p* > 0.05; Figure 1B). Muscle fiber area in the 4 m and 1.5 y Tibetan sheep was significantly lower than other two groups, and muscle fiber diameter increased significantly with increasing age (*p* < 0.05; Figure 1D).

### Meat Quality of Tibetan Sheep at Different Growth Stages

The meat quality of 4 m, 1.5 y, 3.5 y, and 6 y Tibetan sheep are shown in Table 2. L_45min_, L_24h_, and b_45min_ values of 4 m Tibetan sheep were significantly higher than 1.5, 3.5, and 6 y animals (*p* < 0.01), and the a_45min_ value of 3.5 y Tibetan sheep was lowest (*p* < 0.01). Except for b_24h_ of 4 m, a_24h_ and b_24h_ of 6 y were significantly higher than the other groups (*p* < 0.05). The pH_45min_ of 4 m and 3.5 y Tibetan sheep meat was higher than that of 1.5 and 6 y sheep (*p* < 0.01). After being stored at 4°C for 24 h, pH declined in all groups. The IMF content of 1.5 and 3.5 y was higher than that of 4 m and 6 y animals (*p* < 0.01). The shear force of the muscles of 4 m was the lowest, and it gradually increased with increasing age (*p* < 0.01). The drip loss increased with increasing slaughter age (*p* < 0.01), while the cooking loss of 6 y was the lowest among the four groups (*p* < 0.01).

**Table 2:** Effect of different muscle fiber types on meat quality for Tibetan sheep slaughtered at different ages.

**Item**	**4 m**	**1.5 y**	**3.5 y**	**6 y**	***p*** **-Values**
L_45min_	33.78 ± 1.42^a^	26.54 ± 1.46^b^	26.45 ± 1.59^b^	26.97 ± 2.44^b^	<0.001
a_45min_	20.25 ± 0.99^a^	19.39 ± 1.64^a^	17.85 ± 1.85^b^	19.27 ± 1.54^a^	<0.001
b_45min_	8.81 ± 1.13^a^	6.98 ± 0.66^b^	7.56 ± 2.25^b^	6.90 ± 0.99^b^	<0.001
L_24h_	36.16 ± 2.15^a^	30.24 ± 2.66^b^	27.55 ± 3.05^c^	29.96 ± 4.32^b^	<0.001
a_24h_	20.21 ± 1.43^b^	20.02 ± 1.43^b^	19.07 ± 1.57^b^	22.59 ± 2.86^a^	<0.001
b_24h_	9.64 ± 1.41^ab^	9.06 ± 0.89^b^	8.42 ± 2.63^b^	10.88 ± 2.27^a^	0.002
pH_45min_	6.77 ± 0.10^a^	6.63 ± 0.08^b^	6.74 ± 0.10^a^	6.50 ± 0.13^c^	<0.001
pH_24h_	5.44 ± 0.06^a^	5.48 ± 0.25^a^	5.35 ± 0.06^b^	5.25 ± 0.06^c^	<0.001
IMF (%)	1.60 ± 0.32^b^	2.46 ± 0.49^a^	2.36 ± 0.39^a^	1.63 ± 0.26^b^	<0.001
Shear force (*N*)	26.87 ± 3.17^d^	42.86 ± 3.64^c^	50.77 ± 4.18^b^	59.96 ± 3.85^a^	<0.001
Drip loss (%)	2.30 ± 0.14^c^	2.57 ± 0.34^b^	3.00 ± 0.43^a^	3.25 ± 0.33^a^	<0.001
Cooking loss (%)	39.18 ± 1.05^a^	37.53 ± 0.88^b^	38.18 ± 1.53^b^	36.50 ± 1.10^c^	<0.001

a−d *Within a row, values with different superscript letters significantly differ (p < 0.05). Values are shown as the mean values ± S.E*. *IMF, intramuscular fat content*. *p-Values refer to comparisons of meat quality between four different slaughter age*.

### Fatty Acid Profile at Different Growth Stages of Tibetan Sheep

The effect of slaughter age on the fatty acid composition of the LT muscle of Tibetan sheep is shown in Table 3. There are 26 fatty acids detected in the LT muscle of Tibetan sheep. The SFA content of 4 m and 1.5 y sheep was lower than the other two groups, and there was no significant difference between 4 m and 1.5 y sheep, while SFA contents increased in 3.5 y Tibetan sheep (*p* = 0.032). The highest content of MUFA, C14:1, C17:1, and C18:2n6t was in 1.5 y Tibetan sheep (*p* < 0.05).

**Table 3:** Effect of slaughter age on the fatty acid profile of LT muscle of Tibetan sheep (g/100 g).

**Fatty acid**	**4 m**	**1.5 y**	**3.5 y**	**6 y**	***p*** **-Values**
C10:0	0.63 ± 0.098	0.58 ± 0.066	0.50 ± 0.086	0.59 ± 0.014	0.912
C12:0	0.58 ± 0.123^b^	0.57 ± 0.062^b^	0.64 ± 0.035^ab^	0.86 ± 0.125^a^	0.101
C14:0	4.12 ± 0.191^a^	2.05 ± 0.019^b^	2.15 ± 0.056^b^	3.49 ± 0.407^a^	0.002
C14:1	0.66 ± 0.250^b^	1.37 ± 0.276^a^	0.58 ± 0.031^b^	0.67 ± 0.041^b^	0.038
C15:0	0.65 ± 0.011	0.55 ± 0.373	0.26 ± 0.069	0.52 ± 0.012	0.339
C16:0	23.61 ± 1.021^a^	18.81 ± 0.011^c^	20.62 ± 0.951*b*^c^	21.00 ± 0.353^b^	0.012
C16:1	1.30 ± 0.005^b^	1.19 ± 0.027^bc^	1.08 ± 0.081^c^	1.50 ± 0.070^a^	0.006
C17:0	0.84 ± 0.018^c^	1.10 ± 0.235^bc^	1.19 ± 0.012^b^	1.82 ± 0.018^a^	0.005
C17:1	0.37 ± 0.015^c^	0.67 ± 0.005^a^	0.49 ± 0.016^b^	0.54 ± 0.030^b^	<0.001
C18:0	15.88 ± 0.105^c^	21.68 ± 0.107^b^	23.85 ± 0.569^a^	21.06 ± 0.281^b^	<0.001
C18:1n9t	3.47 ± 0.158^bc^	3.54 ± 0.412^b^	4.74 ± 0.105^a^	2.58 ± 0.479^c^	0.013
C18:1n9c	34.12 ± 1.409	34.94 ± 0.132	32.25 ± 1.392	34.20 ± 0.694	0.059
C18:2n6t	0.35 ± 0.004^b^	0.54 ± 0.008^a^	0.25 ± 0.008^b^	0.32 ± 0.078^b^	0.007
C18:2n6c	4.76 ± 0.879	4.85 ± 0.428	4.53 ± 0.445	3.39 ± 0.024	0.249
C20:0	0.20 ± 0.075	0.32 ± 0.057	0.24 ± 0.051	0.30 ± 0.071	0.333
C18:3n6	0.10 ± 0.067	0.14 ± 0.013	0.14 ± 0.030	0.10 ± 0.011	0.531
C20:1	2.73 ± 0.053^a^	1.60 ± 0.223^bc^	1.37 ± 0.147^c^	2.29 ± 0.511^ab^	0.028
C18:3n3	0.92 ± 0.155	0.72 ± 0.177	0.80 ± 0.134	0.76 ± 0.108	0.589
C21:0	0.26 ± 0.146	0.31 ± 0.362	0.45 ± 0.128	0.45 ± 0.118	0.759
C20:2	0.18 ± 0.092	0.14 ± 0.061	0.14 ± 0.029	0.14 ± 0.022	0.827
C22:0	0.53 ± 0.025^a^	0.41 ± 0.060^ab^	0.34 ± 0.041^b^	0.52 ± 0.063^a^	0.048
C20:3n6	0.10 ± 0.003	0.14 ± 0.015	0.15 ± 0.018	0.12 ± 0.006	0.062
C22:1n9	0.09 ± 0.102	0.52 ± 0.494	0.38 ± 0.049	0.37 ± 0.006	0.461
C20:4n6	2.34 ± 0.142^a^	1.64 ± 0.313^b^	1.57 ± 0.350^b^	1.15 ± 0.088^b^	0.038
C20:5n3	0.67 ± 0.078	0.79 ± 0.086	0.56 ± 0.194	0.55 ± 0.013	0.259
C22:6n3	0.55 ± 0.020	0.84 ± 0.185	0.73 ± 0.165	0.71 ± 0.064	0.281
SFA	47.29 ± 1.626^b^	46.37 ± 1.040^b^	50.23 ± 0.260^a^	50.61 ± 0.604^a^	0.032
MUFA	42.75 ± 1.478^b^	43.82 ± 0.805^a^	40.90 ± 1.061^c^	42.15 ± 0.303^b^	0.045
PUFA	9.97 ± 3.111	9.81 ± 0.235	8.88 ± 1.321	7.24 ± 0.301	0.180
n-3	2.13 ± 0.214	2.35 ± 0.447	2.09 ± 0.494	2.02 ± 0.186	0.813
n-6	7.65 ± 2.803	7.32 ± 0.151	6.64 ± 0.798	5.09 ± 0.136	0.128

a−c *Within a row, values with different superscript letters differ (p < 0.05). Data are shown as the mean values ± S.E*. *SFA, MUFA, PUFA, and n represent saturated fatty acid, mono unsaturated fatty acid, poly unsaturated fatty acid, and omega, respectively*. *p-Values represent statistical comparisons of fatty acid profiles between the four slaughter ages*.

### Tibetan Sheep Muscle Fiber Types

**Figure 2:**
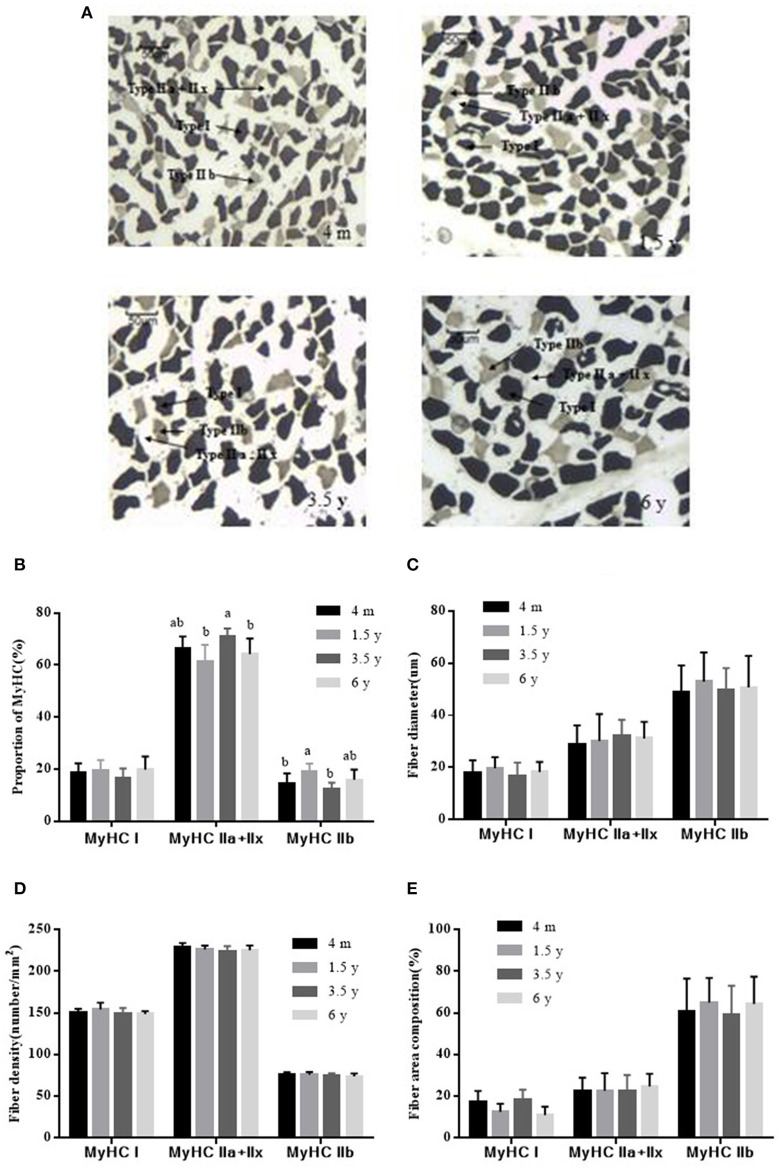
ATPase staining to determine the type of Tibetan sheep muscle fiber (×100). **(A)** Type I fiber was stained dark, the type IIa + IIx fiber was unstained, the type IIb fiber was stained gray. **(B)** Proportions of MyHC I, MyHC IIa + IIx, and MyHC IIb in the LT muscle of Tibetan sheep at different growth stages. **(C)** The diameter of MyHC I, MyHC IIa + IIx, and MyHC IIb at different growth stages. **(D)** Fiber density analysis of MyHC I, MyHC IIa + IIx, and MyHC IIb at different growth stages. **(E)** Fiber area composition of MyHC I, MyHC IIa + IIx, and MyHC IIb at different growth stages. Data are shown as the means ± S.E. Different letters (a,b) indicate significant between different growth stages (*p* < 0.05).

The LT muscle fibers of Tibetan sheep were classified into three types (I, IIa + IIx, and IIb) based on the acid stability of myosin ATPase and the glycolytic rate (Figure 2A). There was no significant difference in the proportion of MyHC I muscle fiber content in the four groups (*p* > 0.05). The maximum proportion of MyHC IIa + IIx in 3.5 y Tibetan sheep was 70.89%, which was higher than that in 1.5 and 6 y Tibetan sheep (*p* < 0.05). The proportion of MyHC IIb was 19.06% at 1.5 y and was higher than that in 4 m and 3.5 y of Tibetan sheep (*p* < 0.05; Figure 2B). There was no significant difference in the diameter of MyHC I, MyHC IIa + IIx, and MyHC IIb fibers between the four growth stages (*p* > 0.05). However, the diameter of each type of muscle fiber was different (*p* < 0.05), with MyHC IIb having the largest diameter (Figure 2C). There was no significant difference in the muscle fiber density of MyHC I, MyHC IIa + IIx, and MyHC IIb (*p* > 0.05); however, MyHC IIa + IIx had the highest density (*p* < 0.01; Figure 2D). There was no significant difference in fiber area composition of MyHC I, MyHC IIa + IIx, and MyHC IIb (*p* > 0.05). MyHC IIb fiber area composition was larger than that of MyHC I and MyHC IIa + IIx (*p* < 0.01; Figure 2E).

### Expression of MyHC Genes at Different Growth Stages

**Figure 3:**
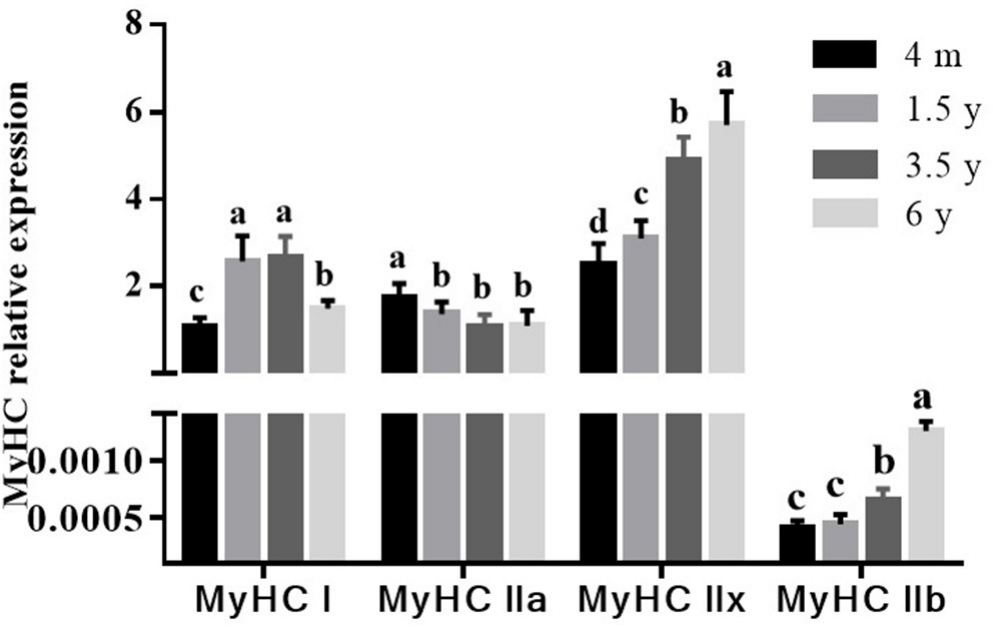
Effect of slaughter age on muscle fiber type-related gene expressions in Tibetan sheep. The mRNA levels were measured by real-time quantitative PCR. The MyHC I, MyHC IIa, MyHC IIx, and MyHC IIb mRNA levels were normalized to the amount of GAPDH mRNA. Data are shown as the means ± S.E. Different letters (a–d) indicate significant differences between different slaughter age (*p* < 0.05).

The MyHC I, MyHC IIa, MyHC IIx, and MyHC IIb mRNA expressions were analyzed by RT-qPCR. As shown in Figure 3, the MyHC I mRNA was the lowest at 4 m (*p* < 0.05) and then increased with age, reaching the highest level at 3.5 years. The MyHC IIa mRNA decreased with increasing age while the MyHC IIx and MyHC IIb mRNA were the least in 4 m Tibetan sheep and gradually increased with age (*p* < 0.05). The MyHC IIb mRNA was lower than MyHC I, MyHC IIa, and MyHC IIx in the LT muscle of Tibetan sheep (*p* < 0.05).

### Correlation Analysis

Positive correlations were found between L_45min_ and MyHC IIa + IIx type muscle fiber density (0.333, *p* < 0.01) and MyHC IIa expression (0.602, *p* < 0.01; Table 4). Negative correlations were found between the L_45min_ value and MyHC I expression (−0.669, *p* < 0.01), MyHC IIx expression (−0.561, *p* < 0.01), and MyHC IIb expression (−0.378, *p* < 0.01). A positive correlation was found between the shear force and MyHC IIb diameter (0.265, *p* < 0.05), and negative correlations between shear force and MyHC IIa + IIx type muscle fiber density (−0.261, *p* < 0.05), MyHC IIb density (−0.352, *p* < 0.01), MyHC I expression (−0.321, *p* < 0.01), and MyHC IIa expression (−0.735, *p* < 0.01), with positive correlations between shear force and MyHC IIx expression (0.862, *p* < 0.01), MyHC IIb expression (0.797, *p* < 0.01), and fiber diameter (0.707, *p* < 0.01). A positive correlation was found between the IMF and MyHC I expression (0.692, *p* < 0.01), and a negative correlation was found between IMF and MyHC IIb expression (−0.327, *p* < 0.01).

**Table 4:** Pearson correlations (two-tailed test, *N* = 72) between various indicators.

	**A**	**B**	**C**	**D**	**E**	**F**	**G**	**H**	**I**	**J**	**K**	**L**	**M**	**N**	**O**	**P**	**Q**	**R**	**S**	**T**	**U**	**V**	**W**
A	1	0.414**	0.531**	0.768**	−0.012	0.125	−0.724**	0.309**	0.204	−0.420**	−0.157	0.499**	0.009	−0.093	−0.250*	0.006	0.333**	0.199	−0.669**	0.602**	−0.561**	−0.378**	−0.616**
B		1	0.311**	0.465**	0.465**	0.340**	−0.302**	−0.042	0.178	−0.146	−0.217	0.224	0.097	0.086	−0.058	−0.013	0.244*	0.168	−0.427**	0.413**	−0.307**	−0.087	−0.221
C			1	0.389**	0.110	0.552**	−0.379**	0.348**	0.059	−0.119	−0.052	0.369**	−0.077	0.240*	−0.053	0.257*	0.176	0.057	−0.283*	0.326**	−0.295*	−0.262*	−0.393**
D				1	0.203	0.345**	−0.586**	0.167	0.119	−0.372**	−0.242*	0.365**	0.051	0.000	−0.028	−0.057	0.279*	0.171	−0.565**	0.453**	−0.531**	−0.295*	−0.566**
E					1	0.728**	0.268*	−0.344**	−0.130	−0.209	0.233*	−0.338**	0.005	0.172	0.130	−0.007	0.105	0.013	−0.333**	0.009	0.261*	0.464**	0.087
F						1	0.140	−0.208	−0.120	−0.247*	0.162	−0.181	−0.015	0.282*	0.165	0.159	0.035	−0.032	−0.313**	−0.005	0.153	0.312**	−0.092
G							1	−0.513**	−0.483**	0.120	0.582**	−0.592**	−0.033	0.049	0.265*	−0.056	−0.261*	−0.352**	−0.321**	−0.735**	0.862**	0.797**	0.707**
H								1	0.185	0.096	−0.232*	0.479**	−0.209	0.079	−0.057	0.045	−0.022	0.152	0.031	0.334**	−0.468**	−0.627**	−0.319**
I									1	0.272*	−0.525**	0.285*	0.244*	−0.080	−0.218	0.137	0.301*	0.369**	0.084	0.399**	−0.493**	−0.535**	−0.362**
J										1	−0.336**	−0.083	0.098	0.128	−0.127	0.139	−0.180	0.066	0.692**	−0.152	−0.063	−0.327**	0.053
K											1	−0.231	−0.251*	0.106	0.118	−0.356**	−0.120	−0.296*	−0.174	−0.455**	0.785**	0.738**	0.535**
L												1	−0.195	−0.081	−0.150	−0.025	0.154	0.221	−0.108	0.445**	−0.517**	−0.579**	−0.449**
M													1	−0.182	0.000	−0.107	0.013	0.082	0.044	−0.012	−0.132	−0.026	−0.055
N														1	0.061	0.039	0.020	0.085	0.036	−0.007	0.036	0.038	−0.014
O															1	−0.110	−0.081	−0.511**	0.009	−0.151	0.191	0.269*	0.160
P																1	0.146	−0.069	0.098	0.131	−0.236*	−0.190	−0.111
Q																	1	0.086	−0.266*	0.232*	−0.309**	−0.161	−0.245*
R																		1	0.037	0.164	−0.349**	−0.344**	−0.328**
S																			1	−0.430**	0.158	−0.190	0.231
T																				1	−0.696**	−0.520**	−0.579**
U																					1	0.841**	0.707**
V																						1	0.654**
W																							1

*A, B, C, D, E, F, G, H, I, J, K, L, M, N, O, P, Q, R, S, T, U, V, and W, respectively, represent L_45min_, a_45min_, b_45min_, L_24h_, a_24h_, b_24h_, shear force, pH_45min_, pH_24h_, IMF, drip loss, cooking loss, fiber diameter of MyHC I, fiber diameter of MyHC IIa + IIx, fiber diameter of MyHC IIb, fiber density of MyHC I, fiber density of MyHC IIa + IIx, fiber density of MyHC IIb, mRNA expression of MyHC I, mRNA expression of MyHC IIa, mRNA expression of MyHC IIx, and mRNA expression of MyHC IIb, fiber diameter*.

* *Correlation is significant at P < 0.05*,

** *Correlation is significant at P < 0.01*.

## Discussion

Sheep meat is an important source of animal protein across the world and is popular for its leanness, lack of fat, tenderness, juiciness, and nutrition (31). Tibetan sheep lives on the Qinghai-Tibet Plateau in China, is famous for its individual flavor, palatability, and nutrition. Meat color is one of the most important physical indicator of meat quality. In this study, the meat color of L_45min_ in Tibetan sheep was the highest in the 4 m group and significantly higher than that of other age groups. This could be because 4 m meat has more moisture and a greater myoglobin content with more type I muscle fiber (32). However, some studies have found that the *L*-value of the meat gradually increased with animal age (33). The a value and b value of the 4 m group were higher compared to the other groups, similar to the results of Warner et al. (34) but contrary to the results of Gardner et al. (5). In general, muscle fiber types at birth are composed of oxidative fibers (35). Under a high-altitude environment with lower temperature and thinner oxygen level, there were more type I muscle fibers and myoglobin content in young animals, which would result in a higher a value (36). In this study, the b value of the 4 m Tibetan sheep was highest, contrary to the results of Mashele et al. (37). After being stored at 4°C for 24 h, the color value of Tibetan sheep meat was increased.

The glycogen content gradually decreased post-mortem, glycogen degraded into lactic acid by glycolysis until the activity of glycolytic enzymes was inhibited, which resulted in pH value continued decline. In this study, the pH_45min_ of Tibetan sheep meat post-mortem was close to neutral, and the 4 m group was the highest, and there were more slow-twitch fibers in 4 m Tibetan sheep meat. Bertol et al. found that pork pH_45min_ was related to the total amount of muscle fibers present, and that pig meat with more fibers had a smaller fiber size, higher pH_45min_, and lower drip loss (38). The pH_24h_ value showed decline from 1.5 to 6 y; pH_24h_ value was lowest in 6 y Tibetan sheep. It is generally believed that the rate and extent of pH decline is mainly related to glycolysis post-mortem, with glycogen degraded into lactic acid and H^+^. Previous studies also found that there was a difference of the rate of glycolysis post-mortem between different muscle fibers types, which influence the meat quality by changing the speed and degree of pH decline (39). The more glycolytic fibers, the more the rate of glycolysis, which resulted in the accumulation of lactic acid, and the pH declined rapidly, finally influencing the meat quality (40). Therefore, the percentage of type IIB fibers in sheep meat was negatively correlated with pH value (18). Muscles harboring higher percentages of fast-twitch fiber tend to have a more rapid pH decline than muscles with a higher percentage of slow-twitch fiber (41).

IMF content is an important factor that influences meat quality, which is affected by genetic and environmental factors such as genotype, gender, feeding system, and age. IMF content is positively correlated with tenderness (15). A previous study using Sudan Black B and Oil Red O for histochemical staining showed that all type I fibers contained neutral lipids while IIA and IIB fibers contained only 26 and 1%, respectively (42). Therefore, the percentage of type I fibers was positively correlated with the IMF content of cattle meat (43). Type I muscle fibers are to the benefit of juiciness and flavor, while type IIB fiber content is often associated with harder meat. There is a consistent correlation between type I muscle fiber and meat tenderness (15). In this study, the IMF content in the 1.5 and 3.5 y Tibetan sheep meat was highest. There were more mitochondria and myoglobin of type I muscle fibers on the electron transfer chain. Kim et al. and Liu et al. found that the more glycolytic muscle fiber types and the less oxidized muscle fiber types in muscle leads to a rapid pH decline post-mortem, which made white meat (44, 45). The meat quality depends largely on the muscle fiber types. The different slaughter ages of animal influence meat quality by regulating the composition of muscle fibers, and each muscle fiber type content changed with increasing age (46). The shear force is one of the most important physical indicators of meat tenderness. In this study, the shear force showed continued increase with age. Abhijith et al. reported that the shear force of Boer goat meat increased significantly with age (6). Drip loss and cooking loss were associated with tenderness and juiciness. In this study, there was a lower drip loss of 4 m, which was associated with more type I muscle fiber. And similar to results of Turan et al. who found young animal had higher meat quality in terms of tenderness and water holding capacity (47).

Fatty acids are important nutrients for the human body and have important physiological functions. In this study, there were 26 fatty acids detected in the LT muscle of Tibetan sheep, including 10 SFAs, seven MUFAs, and nine PUFAs. MUFA is of great significance for reducing cardiovascular diseases with its function of reducing total cholesterol, enhancing the activity of antioxidant enzymes, reducing blood pressure and blood sugar levels, preventing deterioration in memory, and promoting growth and development (48). In this study, the highest MUFA contents were detected in the 1.5 y Tibetan sheep, which then reduced with increasing age. Zhang et al. found that lamb meat had the least MUFA content, complementing the results of our study (1). PUFA plays an important role in stabilizing cell membrane function, regulating gene expression, maintaining cytokine and lipoprotein balance, resisting cardiovascular diseases, and promoting growth and development (49) with a variety of functions within biological systems (1). In this study, PUFA contents showed continued decline with increasing age, which was consistent with the results of Zhang et al. (1). C18:2n6c is an essential fatty acid and the precursor fatty acid for the synthesis of conjugated linoleic acid (CLA). In this study, content of C18:2n6c was highest in the 1.5 y Tibetan sheep. C20:5n3 and C22:6n3 are important PUFA with the function of reducing platelet aggregation and blood lipids, preventing coronary heart disease, improving memory, and preventing brain senescence (50). C20:5n3 and C22:6n3 contents in the 1.5 y group were higher compared to other groups.

In this study, expression differences of these myosin heavy chains were found in the LT muscle of Tibetan sheep at different ages. Correlations were found between muscle fiber type and meat quality, and muscle fiber types largely influence the meat quality of Tibetan sheep. This result was supported by previous studies. Hwang et al. have found that there was a positive correlation between shear force and Type IIb muscle fiber and Type IIb area in Korean native black goat (51). Overall, the complex metabolic characteristics of muscle may play a more important role in determining the meat quality of Tibetan sheep.

## Conclusion

In conclusion, the results of the present study indicated that the meat tenderness and water holding capacity of Tibetan sheep decreased with increasing age, IMF of 1.5 y Tibetan sheep was the highest. Age influenced the ultimate pH and meat color; higher MUFA and some PUFA content were observed in 1.5 y of Tibetan sheep. The above results demonstrated that 1.5 y was a more suitable slaughter age of Tibetan sheep for a healthy human diet. Age influenced the meat quality, which was possibly associated with the transformation of oxidative muscle fiber to glycolytic muscle fiber.

## Data Availability Statement

The original contributions presented in the study are included in the article/Supplementary Material, further inquiries can be directed to the corresponding author/s.

## Ethics Statement

The animal study was reviewed and approved by the Faculty Animal Policy and Welfare Committee of Gansu Agricultural University.

## Author Contributions

GB: data curation and writing—original draft. XL and JH: formal analysis, methodology, and software. JW and BS: investigation, validation, methodology, and software. SL: funding acquisition and writing—review and editing. YL: project administration, supervision, and writing—review and editing. All authors contributed to the article and approved the submitted version.

## Funding

This research was funded by the Basic Research Creative Groups of Gansu Province (17JR5RA137), the Fuxi Young Talents Fund of Gansu Agricultural University (Gaufx-03Y04), the Projects of Gansu Agricultural University (GSAU-ZL-2015-033), and Key R&D Projects in Gansu Province (18YF1WA082).

## Conflict of Interest

The authors declare that the research was conducted in the absence of any commercial or financial relationships that could be construed as a potential conflict of interest.

## Publisher's Note

All claims expressed in this article are solely those of the authors and do not necessarily represent those of their affiliated organizations, or those of the publisher, the editors and the reviewers. Any product that may be evaluated in this article, or claim that may be made by its manufacturer, is not guaranteed or endorsed by the publisher.
